# Supplementary material for: Nosocomial RSV-related In-hospital Mortality in Children <5 Years: A Global Case Series
Source: Pediatr Infect Dis J. 2022 Nov 8;42(1):1–7. doi: 10.1097/INF.0000000000003747 (PMC9891274; doi:10.1097/INF.0000000000003747)
Supplement: Supplementary file 2 [file inf-42-1-s002.pdf]

Nosocomial RSV-related In-hospital Mortality in Children <5 Years: A Global Case Series

**RSV GOLD study group collaborators are:*

Josep Estrada[1], Angela Gentile[2], Maria Florencia Lucion[2], Marcela Echavarria[3], Noelia Reyes[3], Fernando P. Polack[4], Mauricio T. Caballero[4], Annette Alafaci[5,6], Nigel Crawford[5,6], Jenny Thompson[6], Warwick Butt[5,6,7], Nusrat Homaira[8], Adam Jaffe[8], Gemma Saravanos[9], Philip Britton[9], Christoph Binder[10], Angelika Berger[10], Bernhard Resch[11], Fahmida Chowdhury[12], Md. Ariful Islam[12], Senjuti Saha[13], Samir Saha[13], José Gareca Perales[14], Felipe Cotrim de Carvalho[15], Sergio de Andrade Nishioka, on behalf of the Influenza Surveillance Team at the Brazil Ministry of Health[15,16], Maria Tereza da Costa Oliveira[17], Carla Cecília de Freitas Lázaro Emediato[17], Heloisa Giamberardino[18], Jane Melissa Webler[18], Regina Grigolli-Cesar[19,20], Daniel Jarovsky[21], Daniela Gregória Bomfim Prado da Silva[21], Patricia Gomes de Matos Bezerra[22], Maria do Carmo Menezes Bezerra Duarte[22], Fernanda de-Paris[23], Márcia Rosane Pires[24], Sonia M. Raboni[25], Tani Sagna[26], Serge Diagbouga[26], Daniel Garros[27], Michael Hawkes[28], Joanne M. Langley[29], Jill Mutch[29], Kirk Leifso[30], Shaun K. Morris[31], Waison Wong[31], Bosco A. Paes[32], Jesse Papenburg[33], Franco Diaz[34,35], Rodrigo A. Fasce[36], Olga Lopez[37], Ting F. Leung[38], Wei Su[39], Chiang Chun Yuan[39], Zhengde Xie[40], Junhong Ai[40], Evelyn Obando Belalcazar[41], Jaime Fernández-Sarmiento[42], Ledys Izquierdo[43,44,45], Rubén Lasso[43,44,46], Rosalba Pardo-Carrero[43,47], Pablo Vasquez[43,44,48], Eliana Zemanate[43,44,49], Srđan Roglić[50], Thea K. Fischer[51], Sune Rubak[52], Mette Holm[52], Domenica de Mora[53], Alfredo Bruno[53,54], Jenny Ojeda[55], Lida Zamora[55], Erica Dueger[56,173], Terho Heikkinen[57], Gilles Cambonie[58], Jean-Christophe Dubus[59], Melina Messaoudi[60], Juliet Bryant[60], Haoua Tall[61], Bradford D. Gessner[61], Dominique Ploin[62], Come Horvat[62], Nour Hanna[63], Christian Vogelberg[63], Christoph Härtel[64], Andrea Streng[64], Johannes Liese[64], Barbara A. Rath[65,66], Jürgen Seidenberg[67], Geeske Stelljes[67], Evangeline Obodai[68], John Kofi Odoom[68], Irini Eleftheriou[69], Maria Tsolia[69], Vassiliki Papaevangelou[70], Elpiniki Kartsiouni[70], Tapan Dhole[71], Sheetal Verma[71], Rashmi Ranjan Das[72], Ashish Satav[73], Agustinus Sutanto[74], Dario Prais[75,76], Orli Megged[77], Francesca Tortora[78], Fabrizio Chiusolo[78], Giovanni Gabutti[79], Kazuki Iio[80], Satoshi Kusuda[81], Hitoshi Oshitani[82], Hisato Ito[83], Najwa Khuri-Bulos[84], Loai Saadah[85], Iman Basheti[85], Sandra S. Chaves[86], Gideon Emukule[86], Patrick K. Munywoki[87,88], D. James Nokes[87,89], Grieven Paul Otieno[87,89], Ieva Silina[90], Abdulla Alfraij[91], Mohammad Alghounaim[92], Ghassan Dbaibo[93], Rima Hanna-Wakim[93], Yoke-Fun Chan[94], Jamal I-Ching Sam[94], Teck-Hock Toh[95], Jeffrey Soon-Yit Lee[95], David Pace[96], Socorro P Lupisan[97], Marilla G. Lucero[97], Daniel E. Noyola[98], Andreu Comas-García[98], Túfaria Mussá[99], Brigitte Buiteman[100], Diederick E. Grobbee[101,102], Rosalie Linssen[103], Namrata Prasad[104], José F. Sánchez[105], Uzma Bashir Aamir[106], Qalab Abbas[107], Abdul Momin Kazi[107], Sidra Asif Khan[107], Rodrigo DeAntonio[108,109], Xavier Saez-Llorens[108,110], Juana del Valle-Mendoza[111], Wojciech Feleszko[112], Karolina Dumycz[112], Sara Cristina de Tavares Ferreira[113], Ana Beatriz de Sousa Pereira Luzio Vaz[113], Mohammed Ahmed Abdullah Qashnon[114], Khalid Alansari[114,115,116,117], Eun Hwa Choi[118], Yae-Jean Kim[119], Eun Lee[120], Eun-Ae Yang[121], Hyun Mi Kang[121], Kirill Stolyarov[122], Thoon Koh Cheng[123], Kee Thai Yeo[124], Chee Fu Yung[123,125,126], Stefan Grosek[127], Marko Pokorn[127], Cheryl Cohen[128,129], Shabir A. Madhi[130], Michelle J. Groome[130], Jocelyn Moyes[129], Marietjie Venter[131], Adele Visser[132], Cristina O'Callaghan-Gordo[133], Quique Bassat[133,134,135,136,137], Cristina Calvo[138], Xavier Carbonell-Estrany[139], Francisco J. Elola[140], Manuel Sánchez Luna[140], Rosa Rodriguez Fernandez[141], Cristian Launes[142,143], Carmen Muñoz-Almagro[142,143,144], J.A.A. Sampath Jayaweera[145], Martin W. Weber[146], Joachim Luthander[147], Ulrich Heininger[148], Daniel Trachsel[148], Claudia E. Kuehni[149], Cristina Ardura-Garcia[149], Chia-Yu Chi[150], Hsin Chi[151], Shuenn-Nan Chiu[152], Jou-Kou Wang[152], Yhu-Chering

Huang[153], Piyarat Suntarattiwong[154], Somsak Thamthitiwat[155], Nasamon Wanlapakorn[156], Aida Borgi[157], Ahmed Ayari[157], Imen Bel Hadj[158], Khadija Boussetta[158], Benan Bayrakci[159], Esra Koçkuzu[159], Muhterem Duyu[160], Sule Gökçe[161], Aykut Eşki[162], Tanil Kendirli[163], Emrah Gün[163], Edward A. Goka[164], Simon Nadel[165], Marwa Ghazaly[165], Kentigern Thorburn[166,167], Paul S. McNamara[166,167], Soledad Menta[168,169], Nicolás Monteverde[168,170], Sebastián González-Dambrauskas[169,171,172], Dianna M. Blau[173], Katherine Horton[173], Robert F. Breiman[174], Andrea Buchwald[175], Helen Chu[176], Leah Forman[177], Christopher J. Gill[177], Lawrence Mwananyanda[177], Aubree Gordon[178], Natasha Halasa[179], Danielle Hessong[180], Diego Raul Hijano[181], Kim J. Allison[181], Matthew S. Kelly[182], Veena Kumar[183], Asuncion Mejias[184,185], Octavio Ramilo[184,185], Katherine O'Brien, on behalf of the PERCH Study Group[186], Saad B. Omer[187], Thyyar Ravindranath[188], Steven L. Shein[189], Ananya Parlapalli[189], Eric A F Simões[190], Michael C Spaeder[191], Pham Thi Minh Hong[192], Tran Anh Tuan[193], Mohammed Al Amad[194], Abdul Wahed Al Serouri[194]

1. SAAS, Escaldes Engordany, Andorra
2. Department of Epidemiology, Ricardo Gutiérrez Children's Hospital, Buenos Aires, Argentina
3. CEMIC University Hospital, Buenos Aires, Argentina
4. Fundación Infant, Buenos Aires, Argentina. Consejo Nacional de Investigaciones Cientificas y Técnicas (CONICET), Argentina.
5. Murdoch Children's Research Institute, Melbourne, Australia
6. The Royal Children's Hospital, Melbourne, Australia
7. Department of Paediatrics, University of Melbourne, Melbourne, Australia
8. University of New South Wales, Sydney, Australia
9. The University of Sydney Children's Hospital Westmead Clinical School, Sydney, Australia
10. Department of Pediatrics and Adolescent Medicine, Comprehensive Center for Pediatrics, Medical University Vienna, Vienna, Austria
11. Medical University of Graz, Graz, Austria
12. Infectious Diseases Division, International Centre for Diarrhoeal Disease Research, Bangladesh (icddr,b), Dhaka, Bangladesh
13. Dhaka Shishu Hospital, Dhaka, Bangladesh
14. Centro de Pediatria Especializada "CRECER", Santa Cruz de la Sierra, Bolivia
15. Department of Transmissable Diseases, Ministry of Health, Brasília, Brazil
16. Instituto Oswaldo Cruz-Fiocruz, Rio de Janeiro, Brazil
17. Health Secretariat of the City of Belo Horizonte, Belo Horizonte, Brazil
18. Hospital Pequeno Principe, Curitiba, Brazil
19. Hospital Infantil Sabará, São Paulo, Brazil
20. Red Colaborativa Pediátrica de Latinoamérica (LARed Network), São Paulo, Brazil
21. Santa Casa de São Paulo, São Paulo, Brazil
22. Instituto de Medicina Integral Prof. Fernando Figueira (IMIP), Recife, Brazil
23. Transplant Immunology and Personalized Medicine Unit, Laboratory Diagnostics Service, Hospital de Clínicas de Porto Alegre, Porto Alegre, Brazil
24. Infection Control Commission, Hospital de Clínicas de Porto Alegre, Porto Alegre, Brazil
25. Universidade Federal do Paraná, Paraná, Brazil

26. Institut de Recherche en Sciences de la Santé (IRSS), Bobo-Dioulasso, Burkina Faso
27. Stollery Children's Hospital, Edmonton, Canada
28. University of Alberta, Alberta, Canada
29. Canadian Center for Vaccinology (Dalhousie University, IWK Health and NS Health), Halifax, Canada
30. Kingston General Hospital, Kingston, Canada
31. Division of Infectious Diseases, The Hospital for Sick Children, Toronto, Canada
32. Neonatal Division, Department of Pediatrics, McMaster University, Hamilton, Canada
33. Montreal Children's Hospital, McGill University Health Centre, Montreal, Canada
34. Hospital La Florida Dra. Eloísa Díaz, Santiago, Chile
35. Red Colaborativa Pediátrica de Latinoamérica (LARed Network), Santiago, Chile
36. Public Health Institute, Santiago, Chile
37. Hospital Dr. Ernesto Torres Galdames, Iquique, Chile
38. Department of Paediatrics, Faculty of Medicine and Chinese University of Hong Kong-University Medical Center Utrecht Joint Research Laboratory of Respiratory Virus and Immunobiology, Chinese University of Hong Kong, Hong Kong, China
39. CanAm International Medical Center, Guang Zhou, China
40. Beijing Children Hospital, Beijing, China
41. Instituto Roosevelt, Bogotá, Colombia
42. Universidad de La Sabana, Fundación Cardioinfantil-Instituto de Cardiología, Bogotá, Colombia
43. Red Colaborativa Pediátrica de Latinoamérica (LARed Network), Bogotá, Colombia
44. BACON, Bogotá, Colombia
45. Hospital Militar Central, Bogotá, Colombia
46. Fundación Valle de Lili, Cali, Colombia
47. Clínica Infantil Colsubsidio, Bogotá, Colombia
48. Hospital de San José, Bogotá, Colombia
49. Hospital Susana López de Valencia E.S.E., Popayán, Colombia
50. Department of Paediatric Infectious Diseases, University Hospital for Infectious Diseases, Zagreb, Croatia
51. University of Copenhagen, Institute of Public Health, Copenhagen, Denmark
52. Danish Center of Pediatric Pulmonology and Allergology, Department of Pediatrics and Adolescents Medicine, Aarhus University Hospital, Aarhus, Denmark
53. Instituto Nacional de Investigacion en Salud Publica, Guayaquil, Ecuador
54. Universidad Agraria del Ecuador, Guayaquil, Ecuador
55. Ministerio de Salud Pública del Ecuador, MSP, Quito, Ecuador
56. Global Disease Detection and Response Program, US Naval Medical Research Unit No. 3, Cairo, Egypt
57. Turku University Hospital and University of Turku, Turku, Finland
58. Montpellier University Hospital Center, Montpellier, France
59. CHU Timone-Enfants, Marseille, France
60. Emerging Pathogens Laboratory - Fondation Merieux, Lyon-Gerland, France
61. Agence de Medecine Preventive, Paris, France; currently with Pfizer, Inc, New York, NY, USA

62. Services de Reanimation et d'Urgences Pediatriques, Hopital Femme Mere Enfant des Hospices Civils de Lyon, Lyon, France
63. Pediatric Department, University Hospital Carl Gustav Carus, Dresden, Germany
64. Department of Pediatrics, University Hospital Würzburg, Würzburg, Germany
65. Vienna Vaccine Safety Initiative, Berlin, Germany
66. University of Bourgogne-Franche-Comté, Besançon, France.
67. University Hospital for Children, Klinikum Oldenburg AöR, Oldenburg, Germany
68. Noguchi Memorial Institute for Medical Research, University of Ghana, Legon, Ghana
69. Children's Hospital `P.& A.Kyriakou`, Athens, Greece
70. Third Department of Pediatrics, National and Kapodistrian University of Athen, ATTIKON Hospital, Athens, Greece
71. King George's Medical University, Lucknow, India
72. All India Institute of Medical Sciences Bhubaneswar (AIIMS), Bhubaneswar, India
73. MAHAN trust (Mahatma Ghandi Tribal Hospital), Kadhava, India
74. West Nusa Tenggara Provincial Government, Lombok, Indonesia
75. Schneider Children`s Medical Center of Israel, Petah Tikva, Israel
76. Sackler Faculty of Medicine, Tel Aviv University, Tel Aviv, Israel
77. Shaare Zedek Medical Center, Jerusalem, Israel
78. Bambino Gesù Children's Hospital, Rome, Italy
79. University of Ferrara, Department of Medical Sciences, Section of Public Health Medicine, Ferrara, Italy
80. Tokyo Metropolitan Children’s Medical Center, Tokyo, Japan
81. Kyorin University, Tokyo, Japan
82. Department of Virology, Tohoku University Graduate School of Medicine, Sendai, Japan
83. Department of Pediatrics, Nantan General Hospital, Ueno, Yagichoyagi, Nantan-shi, Kyoto, Japan
84. Department of Pediatrics, University of Jordan, Amman, Jordan
85. Applied Science Private University, Amman, Jordan
86. Centers for Disease Control and Prevention (CDC), Influenza Program, Nairobi, Kenya
87. KEMRI Welcome Trust Research Programme, Kilifi, Kenya
88. Department of Nursing Sciences, Pwani University, Kilifi, Kenya
89. University of Warwick, Coventry, UK
90. Children's Clinical University Hospital, Riga, Latvia
91. Pediatric Intensive Care Unit, Department of Pediatrics, Farwaniya Hospital, Kuwait City, Kuwait
92. Department of Pediatrics, Amiri Hospital, Kuwait City, Kuwait
93. American University of Beirut, Beirut, Lebanon
94. University of Malaya, Kuala Lumpur, Malaysia
95. Clinical Research Centre, Sibu Hospital, Ministry of Health Malaysia, Sibu, Sarawak, Malaysia
96. Mater Dei Hospital, Msida, Malta
97. Research Institute for Tropical Medicine, Alabang Muntinlupa City, Metro Manila Philippines

98. Universidad Autonoma de San Luis Potosi, San Luis Potosi, Mexico
99. Universidade Eduardo Mondlane, Maputo, Mozambique
100. University Medical Center Utrecht, Utrecht, Netherlands
101. Julius Global Health, Julius Center for Health Sciences and Primary Care, University Medical Center Utrecht, Utrecht, Netherlands
102. Julius Clinical Science, Zeist, Netherlands
103. Pediatric Intensive Care Unit, Emma Children's Hospital Amsterdam UMC, Amsterdam, The Netherlands
104. Institute of Environmental Science and Research, Auckland, New Zealand
105. Department of Medicine, Hospital Infantil Manuel de Jesus Rivera, Managua, Nicaragua
106. Department of Virology, National Institute of Health, Islamabad, Pakistan
107. Department of Paediatrics and Child Health, Aga Khan University, Karachi, Pakistan
108. Sistema Nacional de Investigación-Secretaría Nacional de Ciencia, Tecnología e Innovación (SNI-SENACYT), Panama City, Panama
109. Centro de Vacunación e Investigación CEVAXIN, Panama City, Panama
110. Hospital Del Niño Dr. José Renán Esquivel, Departamento de Infectología, Panama City, Panama
111. School of Medicine, Research Center of the Faculty of Health Sciences, Universidad Peruana de Ciencias Aplicadas, Lima, Peru
112. Department of Pediatric Respiratory Diseases and Allergy, Medical University of Warsaw, Warsaw, Poland
113. Department of Pediatrics Hospital Dona Estefânia, Centro Hospitalar Universitário de Lisboa Central, Lisbon, Portugal
114. Department of Pediatrics, Division of Pediatric Emergency Medicine, Hamad Medical Corporation, Doha, Qatar
115. Department of Emergency Medicine, Sidra Medicine, Doha, Qatar
116. Weill Cornell Medicine, Doha, Qatar
117. Clinical Department, College of Medicine, QU Health, Qatar University, Doha, Qatar
118. Seoul National University College of Medicine, Seoul, South Korea
119. Sungkyunkwan University School of Medicine, Seoul, South Korea
120. Chonnam National University Hospital, Gwangju, South Korea
121. Department of Pediatrics, College of Medicine, The Catholic University of Korea, Seoul, South Korea
122. WHO Russian Federation, Leningrad, Russian Federation
123. Infectious Diseases Service, KK Women's and Children's Hospital, Singapore
124. Department of Neonatology, KK Women's & Children's Hospital, Singapore, Singapore
125. Duke-NUS Medical School, Singapore, Singapore
126. Lee Kong Chian School of Medicine, Imperial College, NTU Singapore
127. UMC Ljubljana, Ljubljana, Slovenia
128. School of Public Health, Faculty of Health Science, University of the Witwatersrand, Johannesburg, South Africa

129. Centre for Respiratory Diseases and Meningitis, National Institute for Communicable Diseases, Johannesburg, South Africa
130. Medical Research Council: Respiratory and Meningeal Pathogens Research Unit and Department of Science and Technology/National Research Foundation: Vaccine Preventable Diseases, University of the Witwatersrand, Johannesburg, South Africa
131. University of Pretoria, Pretoria, South Africa
132. Department of Philosophy, Practical and Systematic Theology, School of Humanities, University of South Africa, Pretoria, South Africa
133. ISGlobal, Hospital Clínic-Universitat de Barcelona, Barcelona, Spain
134. ICREA, Catalan Institution for Research and Advanced Studies, Barcelona, Spain
135. Pediatrics Department, Hospital Sant Joan de Déu, Universitat de Barcelona, Esplugues, Barcelona, Spain
136. Centro de Investigação em Saúde de Manhiça (CISM), Maputo, Mozambique
137. Consorcio de Investigación Biomédica en Red de Epidemiología y Salud Pública (CIBERESP), Madrid, Spain
138. Hospital La Paz. IdiPAZ Foundation, CIBER Infectious Diseases (ISCIII), Translational Research Network in Pediatric Infectious Diseases (RITIP), Madrid, Spain
139. IRIS group Coordinator, Hospital Clinic, Institut d'Investigacions Biomediques August Pi Suñer (IDIBAPS), Barcelona, Spain
140. Fundación Instituto para la Mejora de la Asistencia Sanitaria / Research Institute Gregorio Marañón, Madrid, Spain
141. Hospital Gregorio Marañón, Madrid, Spain
142. Institut de Recerca Pediátrica Sant Joan de Déu, Hospital Sant Joan de Déu, Barcelona, Spain
143. Epidemiology and Public Health Networking Biomedical Research Centre (CIBERESP), Madrid, Spain
144. Department of Medicine, Universitat Internacional de Cataluñya, Barcelona, Spain
145. Department of Microbiology, Faculty of Medical and Allied Sciences, Rajarata University of Sri Lanka, Saliyapura, Sri Lanka
146. Department of Child and Adolescent Health and Development, World Health Organization, Geneva, Switzerland
147. Astrid Lindgren Children's Hospital, Karolinska University Hospital, Solna, Sweden
148. University of Basel Children's Hospital, Basel, Switzerland
149. Institute of Social and Preventive Medicine, University of Bern, Bern, Switzerland
150. National Health Research Institutes, Zhunan, Taiwan
151. Department of Pediatric Infectious Disease, MacKay Children's Hospital, Taipei, Taiwan
152. Department of Pediatrics, National Taiwan University Hospital, Taipei, Taiwan
153. Chang Gung Memorial Hospital, Taoyuan City, Taiwan
154. Queen Sirikit National Institute of Child Health, Bangkok, Thailand
155. Division of Global Health Protection, Thailand Ministry of Public Health-US Centers for Disease Control and Prevention Collaboration, Nonthaburi, Thailand
156. Faculty of Medicine, Chulalongkorn University, Bangkok, Thailand
157. Hôpital d'enfants Béchir Hamza, Tunis, Tunisia

158. Bechir Hamza Children's Hospital, Tunis, Tunisia
159. Department of Pediatric Intensive Care Medicine, Hacettepe University. Life Support Practice and Research Center, Ankara, Turkey
160. Istanbul Medeniyet University, Goztepe Training and Research Hospital, Istanbul, Turkey
161. Ege University Medical Faculty, Izmir, Turkey
162. Ege University Children's Hospital, Izmir, Turkey
163. Department of Pediatric Intensive Care, Ankara University School of Medicine, Ankara, Turkey
164. School of Health and Related Research, University of Sheffield, Sheffield, UK
165. Saint Mary's Hospital, London, UK
166. Department of Paediatric Intensive Care, Alder Hey Children's Hospital, Liverpool, UK
167. Department of Child Health (University of Liverpool), Institute in the Park, Alder Hey Children's Hospital, Liverpool, UK
168. Hospital Tacuarembó, Tacuarembó, Uruguay
169. Red Colaborativa Pediátrica de Latinoamérica (LARed Network), Montevideo, Uruguay
170. Médica Uruguaya, Montevideo, Uruguay
171. Cuidados Intensivos Pediátricos Especializados (CIPe), Casa de Galicia, Montevideo, Uruguay
172. Facultad de Medicina, Unidad de Cuidados Intensivos de Niños del Centro Hospitalario Pereira Rossell (UCIN-CHPR), Universidad de la República, Montevideo, Uruguay
173. Centers for Disease Control (CDC) and Prevention, Atlanta, USA
174. Emory University, Global Health Institute, Atlanta, USA
175. University of Maryland Baltimore, Baltimore, USA
176. University of Washington, Seattle, USA
177. Boston University School of Public Health, Boston, USA
178. Department of Epidemiology, School of Public Health, University of Michigan, Ann Arbor, USA
179. Vanderbilt University Medical Center, Nashville, USA
180. Children's Hospital Colorado, Colorado, USA
181. St Jude Children's Research Hospital, Memphis, USA
182. Division of Pediatric Infectious Diseases, Duke University, Durham, USA
183. AstraZeneca, Hershey, USA
184. Department of Pediatrics, Division of Infectious Diseases, Ohio State University, Columbus, USA
185. Center for Vaccines and Immunity at Nationwide Children's Hospital, Ohio State University, Columbus, USA
186. International Vaccine Access Center, Johns Hopkins Bloomberg School of Public Health, Baltimore, USA
187. Yale Institute for Global Health, New Haven, USA
188. Morgan Stanley Children's Hospital of New York-Presbyterian, New York, USA
189. Rainbow Babies and Children's Hospital, Cleveland, USA

190. Department of Pediatrics and Center for Global Health, University of Colorado, Aurora, USA
191. Division of Pediatric Critical Care, University of Virginia School of Medicine, Charlottesville, USA
192. Department of Pediatrics, Faculty of Medicine, University of Medicine and Pharmacy at Ho Chi Minh city, and Respiratory Department, Children’s Hospital No2, Ho Chi Minh city, Vietnam
193. Respiratory department, Children's Hospital No1, Ho Chi Minh City, Vietnam
194. Yemen-FETP, Sana'a, Yemen
